# Antidiabetic Effects of *Carassius auratus* Complex Formula in High Fat Diet Combined Streptozotocin-Induced Diabetic Mice

**DOI:** 10.1155/2014/628473

**Published:** 2014-01-08

**Authors:** Zhi-Hong Wang, Cheng-Chin Hsu, Hui-Hsuan Lin, Jing-Hsien Chen

**Affiliations:** ^1^Medical Center of Aging Research, China Medical University Hospital, No. 91, Hsueh-Shih Road, Taichung 40402, Taiwan; ^2^School of Nutrition, Chung Shan Medical University, No. 110, Sec. 1, Chien Kuo North Road, Taichung, 40201, Taiwan; ^3^Department of Medical Research, Chung Shan Medical University Hospital, No. 110, Sec. 1, Chien Kuo North Road, Taichung 40201, Taiwan; ^4^School of Medical Laboratory and Biotechnology, Chung Shan Medical University, No. 110, Sec. 1, Chien Kuo North Road, Taichung 40201, Taiwan

## Abstract

*Carassius auratus* complex formula, including *Carassius auratus*, Rhizoma dioscoreae, *Lycium chinense*, and *Rehmannia glutinosa* Libosch, is a combination prescription of traditional Chinese medicine, which has always been used to treat diabetes mellitus in ancient China. In this study, we provided experimental evidence for the use of *Carassius auratus* complex formula in the treatment of high fat diet combined streptozotocin- (STZ-) induced type 2 diabetes. *Carassius auratus* complex formula aqueous extract was prepared and the effects of it on blood glucose, serum insulin, adipose tissue weight, oral glucose tolerance test (OGTT), total cholesterol, and triglyceride (TG) levels in mice were measured. Moreover, adiponectin, TG synthesis related gene expressions, and the inhibitory effect of aldose reductase (AR) were performed to evaluate its antidiabetic effects. After the 8-week treatment, blood glucose, insulin levels, and adipose tissue weight were significantly decreased. OGTT and HOMA-IR index showed improved glucose tolerance. It could also lower plasma TG, TC, and liver TG levels. Furthermore, *Carassius auratus* complex formula could inhibit the activity of AR and restore adiponectin expression in serum. Based on these findings, it is suggested that *Carassius auratus* complex formula possesses potent anti-diabetic effects on high fat diet combined STZ-induced diabetic mice.

## 1. Introduction

Diabetes mellitus is a chronic metabolic disorder which affects people worldwide. By the year 2030, diabetes mellitus is estimated up to about 5% of the world's population (i.e., 366 million people) [1]. More than 90% of diabetic patients account for type 2 diabetes [2]. The characteristic of type 2 diabetes is insulin resistance and glucose intolerance. Therefore, a newer strategy in the treatment of type 2 diabetes is to reduce insulin resistance in peripheral tissue and control of blood glucose level.

Adiponectin and resistin are two kinds of adipose tissue releasing signals with different functions on the control of insulin sensitivity. Previous studies on adiponectin strongly suggest that lower adiponectin levels play an important role in the development of insulin resistance and metabolism disorder related diseases (such as type 2 diabetes and atherosclerosis) [3–5]. Moreover, more evidence suggests a role of resistin in the etiology of both insulin resistance and type 2 diabetes mellitus [6, 7].

Long-term elevated glucose level in the blood likely leads to a variety of diabetic complications such as neuropathy [8], nephropathy [9], and retinopathy [10]. These are partly caused by an increase of oxidative stress. Furthermore, activation of the polyol pathway via the enzyme aldose reductase (AR), which showed an increased activity during hyperglycemia [11], is responsible for diabetic neuropathy and nephropathy [12, 13]. Thus, AR may act as an important therapeutic target in the control of diabetes [14].

In addition, positive net energy balance, resulting from more energy intake and inefficient action of insulin on peripheral tissues, leads to an accumulation of triglyceride in many tissues. Diacylglycerol acyltransferase (DGAT) catalyzes the final step in the biosynthesis of triacylglycerol from diacylglycerol and fatty acyl-CoA. Moreover, the triglyceride content in the tissues was suggested to be closely correlated to the insulin resistance [15].

Current antidiabetic drugs usually have adverse side effects and ineffectiveness against some long-term diabetic complications [16]. Therefore, discovery and development of novel agents for diabetes are still needed. Plants are recognized as a wonderful source for medicines. It is estimated that 1200 species of plants are used as folk medicines for diabetes [17]. Various pharmacological researches of traditional Chinese medicines (TCMs) have clearly demonstrated their biological properties in the treatment of diabetes, such as antihyperglycemia [18], antioxidantive [19], inhibitory activity of AR [20]. *Carassius auratus* complex formula is a combination prescription of four ingredients including *Carassius auratus*, Rhizoma dioscoreae, *Lycium chinense* and *Rehmannia glutinosa* Libosch. In previous study, results showed that Rhizoma dioscoreae, *Lycium chinene* and *Rehmannia glutinosa* Libosch have potent anti-diabetes effects separately [21–23]. In ancient China, *Carassius auratus* complex formula has always been used in folk medicine to treat diabetes, but it still lacks of scientific evidence of its clinical applications.

Therefore, antidiabetic effects of *Carassius auratus* complex formula in high fat diet combined STZ-induced type 2 diabetic mice have been investigated in the present study. All of these results could enhance our understanding regarding the applications of *Carassius auratus* complex formula toward diabetes.

## 2. Materials and Methods

### 2.1. Animal Model

Male Balb/cbyJ mice, 4-5 weeks old, were obtained from the National Laboratory Animal Center (National Science Council, Taipei City, Taiwan). All animals were handled according to the guidelines of the Instituted Animal Care and Use Committee of Chung Shan Medical University (IACUC, CSMU) for the care and use of laboratory animals. Mice were housed on a 12 h light/dark cycle. After adaptation for one week, mice were fed with high fat (60% calories) diet for 2 weeks. Then, diabetes was induced by intraperitoneal injection of streptozotocin (STZ, Sigma, St. Louis, MO, USA) for five days continuously at 40 mg/kg in citrate buffer (0.1 M citric acid, pH 4.5) after a 4 h fasting as described previously [24, 25]. Blood glucose level was monitored on day 10 from the tail vein by using a blood glucose meter (Lifescan Inc. Milpitas, CA, USA). Mice with fasting blood glucose level ≥180 mg/dL were used for this study.

### 2.2. Treatment Protocol

After diabetes was induced, mice were divided into four groups (10 mice per group): diabetic mice with chow diet (DM), or *Carassius auratus* complex formula aqueous extract powder (DM+ low dose formula/DL, DM+ high dose formula/DH). One group of nondiabetic mice with normal chow diet and without STZ injection (Normal) was used for comparison. The powder mixtures of *Carassius auratus* complex formula are manufactured by Everprofit Biotech Inc. (Taichung, Taiwan) following the approved good manufacturing practice (GMP) of Taiwan. The *Carassius auratus* complex formula obtained satisfied herb, heavy metals, general bacteria, fungi, and specific pathogens criteria, which was determined by carrying out the respective confirmation tests, and the final yield from the original dried mixture was 8.3% (w/w). The *Carassius auratus* complex formula consists of *Carassius auratus*, Rhizoma dioscoreae, Lycium chinene, and Rehmannia glutinosa libosch (Table 1). *Carassius auratus* complex formula powder at DL (6.25 mg/kg/day) and DH (62.5 mg/kg/day) was mixed with chow diets.

### 2.3. Experimental Design

After 8-week treatment of *Carassius auratus* complex formula, an oral glucose tolerance test (OGTT) was performed after fasting for 4 h. Blood samples were obtained from the tail vein to monitor blood glucose levels at 0, 30, 60, and 120 min after oral glucose administration 2 g/kg BW. Mice were sacrificed with carbon dioxide. Liver, kidney, and epididymal adipose tissue from each mouse were collected. Blood was also collected, and serum was separated immediately. 0.1 g sample of liver, kidney and epididymal adipose tissue was homogenized on ice in 2 mL phosphate-buffered saline (PBS, pH 7.2). The protein concentration of sample homogenate was determined by the method of Lowry et al. [26] using bovine serum albumin as a standard.

### 2.4. Blood Glucose and Insulin Analysis

The serum glucose level (mg/dL) was measured by a glucose HK kit (Sigma, St Louis, MO, USA). The serum insulin level (*μ*g/L) was measured by a method using a mouse insulin EIA kit (Mercodia AB, Sylveniusgatan 8A, Uppsala, Sweden). Insulin resistance was estimated using the homeostasis model of assessment-insulin resistance (HOMA-IR) formula: (fasting glucose (mmol/L) × fasting insulin (*μ*U/mL)/22.5).

### 2.5. Measurement of Serum and Hepatic Triglyceride and Total Cholesterol Contents

Triglyceride (TG) and total cholesterol (TC) levels (mg/dL) in serum were determined by triglycerides/GB kit and cholesterol/HP kit (Boehringer Mannheim, Germany), respectively. The liver (100 mg) of each mouse was homogenized, total lipids of the liver homogenates were extracted with 12 mL of chloroform and methanol mixture (2 : 1, v/v) according to the method of Folch et al. [27], and the amounts of TG were determined using the same way as described above.

### 2.6. Measurement of Thiobarbituric Acid Reactive Substances (TBARS)

The levels of malondialdehyde (MDA, a marker for lipid peroxidation) were determined as described previously [28]. Briefly, the sample of serum, liver, or kidney was homogenized in Tris-HCl buffer (pH 7.4) using a polytron homogenizer. An aliquot (100 *μ*L) of homogenate was added with a reaction mixture, which included 2% SDS, 20% acetic acid, and 0.7% thiobarbituric acid in capped testing tube. Samples were then incubated at 95°C water bath for 3 h. After incubation, samples were centrifuged at 4000 ×g for 5 min. The supernatants were removed and the absorbance was read at 535 nm. The standard curve was generated with tetramethoxypropane, which yields MDA under similar conditions, for quantification use. The MDA content was expressed as nmol/mg protein.

### 2.7. Activity of Serum and Kidney Aldose Reductase (AR)

Serum and kidney homogenate was centrifuged and the supernatant was used for analysis. The method of Nishinaka and Yabe-Nishimura [29] was used to measure AR activity by monitoring the decrease in absorbance at 340 nm due to NADPH oxidation.

### 2.8. Resistin, DGAT1, and DGAT2 Gene Expression Levels

Real-time PCR was performed to quantify the mRNA expression level of resistin, DGAT1 in adipose tissue, and DGAT2 in liver. Total RNA was extracted using TRIzol reagent (Invitrogen, Life Technologies, Carlsbad, CA, USA) according to the manufacturer's instructions. Total cDNA was obtained by reverse transcription. Specific primers are shown in Table 2 and GAPDH was used as the housekeeping gene to normalize the values obtained for transcripts under examination. The reaction mixture was incubated at 95°C for 10 min and then run for 40 cycles at 95°C for 15 sec and 60°C for 1 min in the ABI Prism 7000 sequence detection system (Applied Biosystems, Foster City, CA, USA).

### 2.9. Measurement of Fructosamine Content

Fructosamine content, short control marker of diabetes, was measured by the change in absorbance resulting from the reduction of nitroblue tetrazolium on a spectrophotometer as described previously [30]. Fructosamine content was expressed as percentage (%) which was compared to control group.

### 2.10. Western Blot Analysis of Adiponectin

Western blot analysis of serum adiponectin was performed. In brief, the blood samples were dissolved in RIPA lysis buffer (50 mM Tris-HCl, pH 7.4, 1% NP-40, 150 mM NaCl, 1 mM EDTA, 1 mM phenylmethylsulfonyl fluoride, and 1 g/mL aprotinin). Aliquots (40 *μ*g) of lysate were resolved on 12% SDS-PAGE. After electrophoresis, the proteins were transferred to PVDF membranes (Millipore). The membranes were blocked with 5% nonfat milk in TBST (20 mM Tris-HCl, pH 7.6, 135 mM NaCl; 0.1% Tween 20). The blots were then incubated with the antibodies of adiponectin (Abcam, Cambridge, UK) and *β*-actin (Santa Cruz, CA, USA). The secondary antibodies were goat anti-rabbit and goat anti-mouse horseradish peroxidase-labeled antibodies. The signals were visualized by ECL Western Blotting Detection Reagent (Millipore, Billerica, MA).

### 2.11. Statistical Analysis

The effect of each measurement was analyzed from 10 mice (*n* = 10). Results were expressed as means ± SD. Statistical analysis was done using one-way analysis of variance, and post hoc comparisons were carried out using Duncan's multiple-range test. A difference between two means was considered statistically significant when *P* < 0.05.

## 3. Results

### 3.1. Effects of *Carassius auratus* Complex Formula on Body Weight, Blood Glucose, Insulin, Adipose Tissue Weight, and OGTT

**Figure 1:**
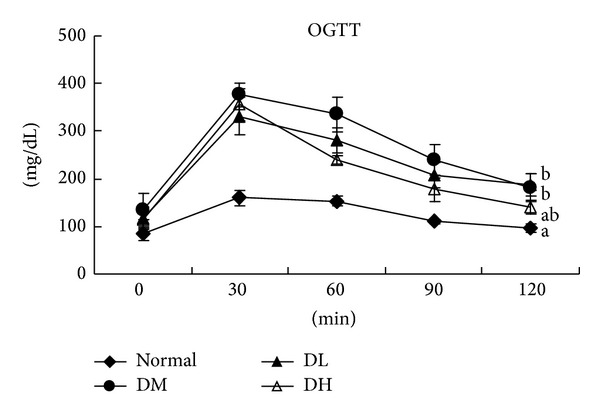
OGTT in normal (Normal), diabetic mice consumed normal diet (DM), or *Carassius auratus* complex formula (DL and DH) at week 8. Values are represented as mean ± SD (*n* = 10).  ^a-b^Means in a time point without a common letter differ, *P* < 0.05.

After *Carassius auratus* complex formula supplement for 8 weeks, the OGTT can be used to evaluate blood glucose homeostasis. As shown in Table 3, *Carassius auratus* complex formula supplement did not significantly decrease body weight gains (*P* > 0.05) but significantly lowered epididymal adipose tissue weight when compared with diabetic control group (*P* < 0.05). Levels of blood glucose and insulin increased after the induction of type 2 diabetes, and so did HOMA-IR index (*P* < 0.05). *Carassius auratus* complex formula treatment significantly reduced blood glucose and insulin levels and HOMA-IR (*P* < 0.05). *Carassius auratus* complex formula treatment also improved oral glucose tolerance (Figure 1, *P* < 0.05).

### 3.2. Effects of *Carassius auratus* Complex Formula on Lipid Profiles and Antioxidantive Status

As shown in Table 4, plasma TG and TC and liver TG levels were higher in diabetic groups when compared with normal group (*P* < 0.05). Treatment of low and high dose of *Carassius auratus* complex formula significantly decreased TG and TC levels in both plasma and liver (*P* < 0.05). Diabetes increased the oxidative stress status as determined by TBARS; *Carassius auratus* complex formula treatment significantly reduced TBARS levels in serum, liver and kidney when compared with diabetic group (*P* < 0.05, Table 5).

### 3.3. Effects of *Carassius auratus* Complex Formula on Aldose Reductase Activity and Serum Fructosamine Level

**Figure 2:**
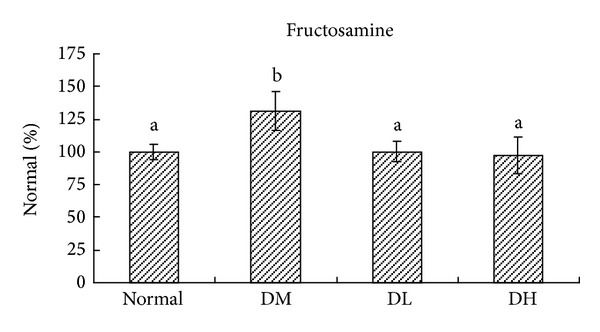
Fructosamine level in normal (Normal), diabetic mice consumed normal diet (DM), or *Carassius auratus* complex formula (DL and DH) at week 8. Values are represented as mean ± SD (*n* = 10).  ^a-b^Means among bars without a common letter differ, *P* < 0.05.

Table 6 and Figure 2 show the AR activity analysis after 8-week treatment. In the *Carassius auratus* complex formula treatment group (both in low and high dose), the activity of AR was apparently suppressed (*P* < 0.05). Similarly, the serum fructosamine level was significantly reduced in *Carassius auratus* complex formula treatment group than diabetic group (*P* < 0.05).

### 3.4. Effects of *Carassius auratus* Complex Formula on Adiponectin, Resistin, DGAT1, and DGAT2 Levels

**Figure 3:**
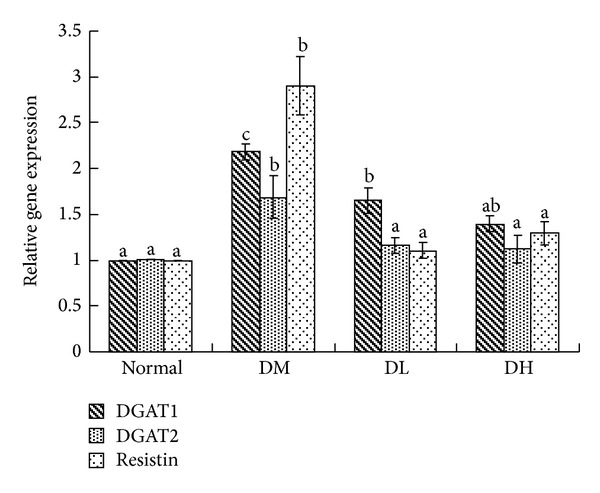
Resistin, DGAT1 and DGAT2 geneexpressionin epididymal adipose tissue and liver of normal (Normal), diabetic mice consumed normal diet (DM) or *Carassius auratus* complex formula (DL and DH) at week 8. Values are represented as mean ± SD (*n* = 10).  ^a–c^Means among bars without a common letter differ, *P* < 0.05.

**Figure 4:**
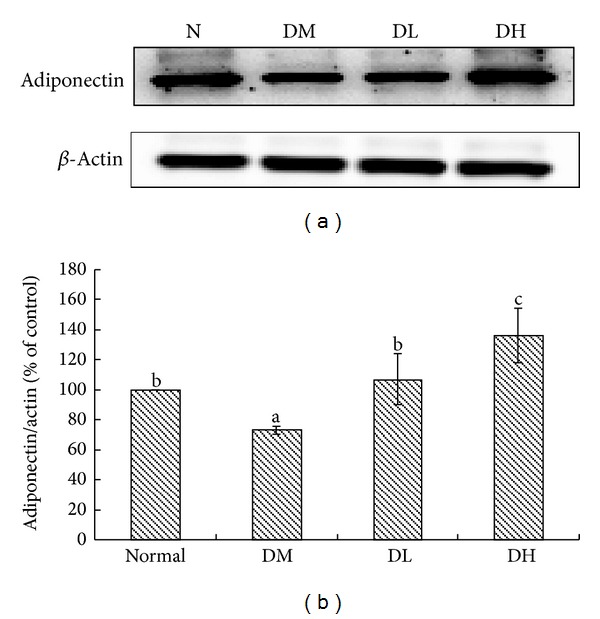
Adiponectin level in serum of normal (N), diabetic mice consumed normal diet (DM), or *Carassius auratus* complex formula (DL and DH) at week 8. Values are represented as mean ± SD (*n* = 10).  ^a-b^Means among bars without a common letter differ, *P* < 0.05.

Figure 3 shows the effect of *Carassius auratus* complex formula on mRNA expression in the liver and adipose tissue. The expression of genes involved in TG synthesis such as DGAT1 and DGAT2 in the *Carassius auratus* complex formula treatment group was significantly lower than that in the diabetic group (*P* < 0.05). For adipose tissue, resistin was significantly downregulated in the *Carassius auratus* complex formula treatment group that in diabetic group (*P* < 0.05). At the same time, the protein expression of adiponectin was significantly restored after the low dose *Carassius auratus* complex formula treatment (*P* < 0.05, Figure 4). Moreover, high dose treatment could increase the protein expression of adiponectin.

## 4. Discussion

It has well been known that type 2 diabetes is a multiorgan disease characterized by impaired insulin sensitivity and altered lipid metabolism and storage [31]. The most common signs seen in diabetes are hypertriglyceridemia, hypercholesterolemia, and TG accumulation in liver and adipose tissue. It is suggested by some previous studies that excess amount of TG accumulation in adipose tissue leads to obesity, and moreover ectopic storage of TG in nonadipose tissue such as liver is associated with insulin resistance and glucose intolerance [32, 33]. In the present study, our results showed that treatment of diabetic mice with *Carassius auratus* complex formula reduced insulin resistance as indicated by OGTT and HOMA-IR index and normalized lipid storages in liver and adipose tissue. In addition, we have also observed that serum TG and TC levels were significantly reduced after 8-week treatment, indicating that *Carassius auratus* complex formula could improve glucose homeostasis and restore abnormal lipid metabolism in type 2 diabetes.

As shown in Table 1, the *Carassius auratus* complex formula is mainly composed of crucian carp, a fish, which may help to act as a good source of protein. Data from an ecologic study suggest that fish intake may play a role in the prevention of type 2 diabetes [34], which is also supported by an animal experiment showing a favorable effect of long-chain omega-3 fatty acids, which are abundant in fish, on insulin resistance [35]. Recent study has indicated that high fish intake was associated with a lower risk of type 2 diabetes in Japanese men [36]. In the present study, our results showed that crucian carp possesses antidiabetic effects and could restore the protein expression of adiponectin, one kind of adipose tissue releasing signals with its function in the control of insulin sensitivity. Together with Rhizoma dioscoreae, Lycium chinene, and Rehmannia glutinosa libosch in formula, it might improve the nutrition status under diabetes condition. Furthermore, previous studies have demonstrated that Rhizoma dioscoreae, Lycium chinene, and Rehmannia glutinosa libosch, respectively, possess anti-insulin resistance, antioxidant, and hypoglycemic activities [21–23]. The evidence cooperatively demonstrated that antidiabetic effects of this formula may be performed by these various components.

Although biosynthesis of TG is essential for normal life physiology, excess amount of TG accumulation results in obesity. The most common type of lipid abnormalities in diabetes is triglycerides accumulation in liver and adipose tissue, and thus it increases insulin resistance. In our study, we showed the phenomenon of reduced DGAT genes expressions in liver and adipose tissue after treatment in mice. This could be a strategy for the treatment of type 2 diabetes.

In previous study, McTernan et al. [37] found that the gene expression of resistin was upregulated in men who had abdominal obesity, indicating that visceral fat deposition is well association with resistin expression and insulin resistance [38]. In our results, we have observed that *Carassius auratus* complex formula treatment significantly downregulated resistin expression in parallel with decreased abdominal adiposity and reduced insulin resistance.

Some evidence revealed that adiponectin is an adipocyte-specific protein, which plays an important role in energy and glucose metabolism [39], and its reduced circulating level is linked to obesity, insulin resistance, and diabetes [40]. Moreover, Yamauchi et al. [41] suggested that adiponectin could rescue insulin resistance via reducing TG contents in liver and skeletal muscles. In accordance with these studies, our results demonstrated that treatment of diabetic mice with *Carassius auratus* complex formula improves the insulin resistance and glucose intolerance via restoring and/or enhancing the circulating level of adiponectin.

Hyperglycemia is frequently found in type 2 diabetes and is often viewed as a risk factor for diabetic complications [42]. Diabetic patients with chronic hyperglycemia lead to an increase in the activity of AR from 3% to 30% and finally complications happen [43, 44]. In this study, treatment of *Carassius auratus* complex formula suppressed the increase of AR compared to diabetic group, as a meanwhile decreasing fructosamine level. The fructosamine is an index of intermediate glucose control (one to three weeks) and valuable screening test for diabetes mellitus [45, 46]. Moreover, long-term hyperglycemia results in increased production of reactive oxygen species (ROS) [47]. In this study, treatment of *Carassius auratus* complex formula reduced serum, liver, and kidney MDA levels, supporting a beneficial effect for type 2 diabetes management.

In conclusion, these results supported that *Carassius auratus* complex formula supplement had effects on reducing visceral fat accumulation, decreasing blood lipids, enhancing insulin sensitivity, reducing oxidative stress, and inhibiting polyol pathway in type 2 diabetic mice.

## Figures and Tables

**Table 1:** Composition of *Carassius auratus* complex formula.

Common name	Scientific name	Composition (%)	Used part
Crucian carp	*Carassius auratus *	88.8	Eviscerated fish
Wolfberry	*Lycium chinense *	4.2	Fruit
Yam	Rhizoma dioscoreae	4.2	Root and stem
Prepared rhizome of adhesive Rehmannia	*Rehmannia glutinosa* Libosch	2.8	Root

**Table 2:** Primer sets used for real-time PCR.

Gene	Forward	Reverse
DGAT1	5′-GGTGCCCTGACAGAGCAGAT-3′	5′-CAGTAAGGCCACAGCTGCTG-3′
DGAT2	5′-GGGTCCAGAAGAAGTTCCAGAAG-3′	5′-CCCAGGTGTCAGAGGAGAAGAG-3′
Resistin	5′-AGACTGCTGTGCCTTCTGGG-3′	5′-CCCTCCTTTTCCTTTTCTTCCTTG-3′
GAPDH	5′-TGTGTCCGTCGTGGATCTGA-3′	5′-TTGCTGTTGAAGTCGCAGGAG-3′

**Table 3:** Body weight (BW), blood glucose (BG), insulin, HOMA-IR index, and epididymal adipose tissue weight of normal (Normal), diabetic mice consuming normal diet (DM), or *Carassius auratus* complex formula (DL and DH) at week 8.

	Normal	DM	DL	DH
BW (g)	30.25 ± 2.59	30.33 ± 1.50	30.29 ± 1.54	29.93 ± 1.67
BG (mg/dL)	142.71 ± 19.54^a^	191.5 ± 28.79^b^	165.63 ± 31.47^a^	153.44 ± 24.84^a^
Insulin (*μ*g/L)	1.14 ± 0.39^a^	2.40 ± 0.72^b^	2.06 ± 0.80^b^	2.15 ± 0.97^b^
HOMA-IR	9.87 ± 3.92^a^	27.53 ± 8.80^c^	20.49 ± 8.51^b^	17.47 ± 7.90^b^
Adipose tissue weight (mg/g BW)	10.25 ± 3.18^a^	15.06 ± 2.48^b^	12.33 ± 3.37^ab^	11.54 ± 4.65^a^

Values are represented as mean ± SD (*n* = 10).

^a–c^Means in a row without a common letter differ, *P* < 0.05.

**Table 4:** Triglycerides (TG) and total cholesterol (TC) levels in plasma and liver of normal (Normal), diabetic mice consuming normal diet (DM), or *Carassius auratus* complex formula (DL and DH) at week 8.

	Normal	DM	DL	DH
Plasma				
TC (mg/dL)	120.79 ± 8.98^a^	145.30 ± 12.24^b^	127.79 ± 11.57^a^	126.66 ± 8.30^a^
TG (mg/dL)	128.76 ± 12.32^b^	153.87 ± 30.71^c^	101.75 ± 25.80^a^	98.76 ± 20.69^a^
Liver				
TG (mg/g protein)	71.26 ± 15.34^a^	136.22 ± 22.19^c^	112.88 ± 29.77^b^	91.69 ± 34.15^a^

Values are represented as mean ± SD (*n* = 10).

^a–c^Means in a row without a common letter differ, *P* < 0.05.

**Table 5:** TBARS levels in serum, liver, and kidney of normal (Normal), diabetic mice consuming normal diet (DM), or *Carassius auratus* complex formula (DL and DH) at week 8.

	Normal	DM	DL	DH
Serum (*μ*M)	0.72 ± 0.18^a^	1.08 ± 0.35^b^	0.67 ± 0.26^a^	0.65 ± 0.22^a^
Liver (nmol/mg protein)	0.16 ± 0.01^a^	0.18 ± 0.02^b^	0.14 ± 0.03^a^	0.14 ± 0.01^a^
Kidney (nmol/mg protein)	0.51 ± 0.06^ab^	0.60 ± 0.03^c^	0.53 ± 0.04^b^	0.48 ± 0.08^a^

Values are represented as mean ± SD (*n* = 10).

^a–c^Means in a row without a common letter differ, *P* < 0.05.

**Table 6:** Aldose reductase activity of normal (Normal), diabetic mice consuming normal diet (DM), or *Carassius auratus* complex formula (DL and DH) at week 8.

	Normal	DM	DL	DH
Serum (nmol/min/mg protein)	1.06 ± 0.45^a^	2.69 ± 0.60^c^	1.77 ± 0.64^b^	1.34 ± 0.51^ab^
Kidney (nmol/min/mg protein)	15.62 ± 1.87^a^	22.06 ± 2.92^b^	16.96 ± 4.34^a^	16.17 ± 3.36^a^

Values are represented as mean ± SD (*n* = 10).

^a–c^Means in a row without a common letter differ, *P* < 0.05.
